# Feasibility of an exercise-nutrition-psychology integrated rehabilitation model based on mobile health and virtual reality for cancer patients: a single-center, single-arm, prospective phase II study

**DOI:** 10.1186/s12904-024-01487-3

**Published:** 2024-06-20

**Authors:** Yuan Qi, Mengjie Wang, Ya Xue, Jingyan Yue, Chunjian Qi, Weihu Shang, Weifen Meng, Wenyu Zhu, Xiaolin Pu, Dongqing Li, Hua Jiang

**Affiliations:** 1 The Affiliated Changzhou No.2 People’s Hospital of Nanjing Medical University, 68 Gehu Middle Road, Wujin District, Changzhou, China; 2 Beijing Ainst Medical Technology Co., Ltd, Beijing, China

**Keywords:** Integrated rehabilitation, Exercise, Nutrition, Psychology, Virtual reality, Mobile health, Quality of life

## Abstract

**Objective:**

Explore the feasibility of a mobile health(mHealth) and virtual reality (VR) based nutrition-exercise-psychology integrated rehabilitation model in Chinese cancer patients.

**Methods:**

We recruited cancer patients in the Oncology department of the Affiliated Changzhou No. 2 People’s Hospital of Nanjing Medical University from October 2022 to April 2023. The rehabilitation program was provided by a team of medical oncologists, dietitians, psychotherapists, and oncology specialist nurses. Participants received standard anti-cancer therapy and integrated intervention including hospitalized group-based exercise classes, at-home physical activity prescription, behavior change education, oral nutrition supplements, and psychological counseling. An effective intervention course includes two consecutive hospitalization and two periods of home-based rehabilitation (8 weeks). Access the feasibility as well as changes in aspects of physical, nutritional, and psychological status.

**Results:**

At the cutoff date of April 2023, the recruitment rate was 75% (123/165). 11.4%patients were lost to follow-up, and 3.25% withdrew halfway. Respectively, the completion rate of nutrition, exercise, and psychology were 85%,55%, and 63%. Nutrition interventions show the highest compliance. The parameters in nutrition, psychology, muscle mass, and quality of life after the rehabilitation showed significant improvements (*P* < .05). There was no significant statistical difference (*P* > .05) in handgrip strength and 6-minute walking speed.

**Conclusion:**

It is feasible to conduct mHealth and VR-based nutrition-exercise-psychology integrated rehabilitation model in Chinese cancer patients. A larger multi-center trial is warranted in the future.

**Trial registration:**

ChiCTR2200065748 Registered 14 November 2022.

**Supplementary Information:**

The online version contains supplementary material available at 10.1186/s12904-024-01487-3.

## Background

Cancer ranks as a leading healthcare issue, with 19.3 million new cases globally in 2020 alone, and 28.4 million new cases projected for 2040 [1]. The cancer itself or the related therapy may lead to emotional, physical, social suffering and elevated levels of psychological distress and depression [2–4]. These negative effects can reduce the compliance of treatments, resulting in decreased overall survival. Therefore, the development of an integrated model is required [5, 6].

Research indicates that interventions such as nutritional guidance, physical activity, and psychological support positively affect patient outcomes [7]. Nutritional interventions can improve patients’ nutritional status and enhance tolerance to oncological treatments; physical activity has beneficial effects on the physical and mental health of cancer patients; and psychological support effectively reduces occurrences of depression and anxiety [8, 9]. However, current research on improving life quality for these patients often involves isolated interventions, lacking studies on the effects of multi-modal, integrated rehabilitation approaches.

Physical activity, a key component of healthy behaviors, benefits cancer prognosis and rehabilitation, alleviates psychological issues like anxiety and depression, and reduces the risk of cancer recurrence and co-morbid cardiovascular diseases [10]. Nonetheless, studies show that awareness and understanding of physical activity among cancer patients are insufficient, with only 30–47% adhering to prescribed exercise regimens in the absence of supervision [11].

Virtual reality (VR) technology immerses participants fully, enhancing sensory activities and compensating for the limitations of traditional methods [12] In recent years, with the rapid advancement of science and technology, various industries have closely integrated, and the concept of “VR + X (application fields)” has been widely applied. This integration has become a driving force for further development across multiple sectors. Particularly, biofeedback technology has emerged as a novel psychological therapy method for restoring physical and mental health. Virtual reality (VR) has been tested in clinical conditions to alleviate anxiety and distress [13], like stroke-related deficits [14], and Parkinson’s disease [15]. Virtual Reality Rehabilitation (VRR) with its entertaining and game-like nature [16, 17] has been proven to improve both adherence rates and training intensity.

In recent years, mobile health (mHealth) has rapidly developed, enabling the creation, monitoring, and evaluation of exercise plans through websites, apps, and WeChat. By providing patients with intelligent and personalized management methods, mHealth feasibly and effectively improves patient health behaviors [18, 19]. It is also a potential way to conduct music therapy (MT) [20] to cope with negative emotions [21]. Currently, mHealth innovation is relatively nascent in cancer care [22].

Current evidence on the feasibility of combined psychology and nutrition interventions in cancer patients is inconclusive, and no trials to date have integrated physical activity management into rehabilitation for this population [23]. Therefore, exploring VR and mHealth-based exercise-nutrition-psychology integrated rehabilitation model is an ideal approach to increase engagement and compliance in cancer patients. The primary aim was to assess the feasibility of this model. Feasibility was defined a priori as at least 70% recruitment, 50% completion in each aspect, and no adverse events. The secondary aim was to explore whether there were changes in aspects of physical, nutritional, and psychological status.

## Materials and methods

### Study decision

This trial was a single-center, single-arm, prospective phase II study conducted by the oncology department of Changzhou No. 2 People’s Hospital. We planned to enroll 100 cancer patients. The inclusion criteria were as follows: (i) Voluntarily participate and sign the informed consent form in writing; (ii) Age ≥ 18 years old, gender is not limited; (iii) malignant tumors clearly diagnosed by pathology and/or cytology; (iv) Estimated hospitalization more than 7 days; (v) Estimated survival ≥ 6 months; (vi) General physical condition (ECOG) 0–2; (vii) Have reading comprehension skills and be able to complete questionnaire. Patients were excluded if they had the following situations: (i) Clinically significant cardiovascular disease, such as heart failure (grade NYHA III-IV), uncontrolled coronary heart disease, cardiomyopathy, uncontrolled arrhythmia, uncontrolled hypertension or history of myocardial infarction within the previous 1 year; (ii)Neurological or psychiatric abnormalities affecting cognitive abilities, including central nervous system metastases; (iii) uncontrolled systemic diseases, such as poorly controlled diabetes; (iv) Mechanical or functional intestinal obstruction. Completion criteria were summarized in Table 1.

**Table 1:** Research program and completion criteria

Sessions	Assessment	Interventions	Completion criteria
Nutrition	NRS-2002 Body composition test	Nutrition counseling guidance; Oral nutrition supplement^a^	(i)Participate at least 2 times nutrition counseling (ii) Follow the7-day dietary suggestion during home rehabilitation. (iii) Complete T1 and T2 assessments
Exercise	6-minute walking test Mobile wearables	Group-based exercise; Home exercise prescription	(i)Complete at least 4 times group-based exercise every hospitalization (ii) Complete > 30 min moderate intensity exercise per day during home rehabilitation (iii) Complete T1 and T2 assessments
Psychology	CFS; DT; HADS; EORTC QLQ-C30 (V3.0)	VR treatment; Music therapy	(i)Complete at least 4 times VR treatment every hospitalization (ii) Complete > 30 min music treatment per day during home rehabilitation (iii) Complete T1 and T2 assessments

The study was designed to carry out with standard cancer therapy as an extra rehabilitation method for cancer patients, provided by a team of medical oncologists, dietitians, and psychologists [24], featuring group-based exercise classes, at-home physical activity prescription, behavior change education, nutritional instruction, oral nutritional supplements, and psychological counseling [25]. The assessments and interventions were shown in Table 1. An effective course included two consecutive hospitalization and two periods of home rehabilitation (8 weeks). Baseline assessments were performed when the patient agreed to enter the study (T1 point). Subsequent reassessments were measured at the third hospitalization (T2 point). During hospitalization, daily compliance was collected by medical staff. During home-based rehabilitation, we use the mHealth app^a^ to follow up to review compliance and modify the program. This study was approved by the Clinical Medical Technology Ethics Committee of Changzhou Second People’s Hospital (Ref: [2022] YLJSA040). All enrolled patients signed informed consent.

### Intervention methods

#### Physical

During hospitalization, patients were ordered to wear mobile wearable devices^b^ (Bluetooth armband or Bluetooth watch) [22], and join the group-based exercise class under the guidance of doctors; Our exercise class contained four phases (Supplement Table 1).

During home-based rehabilitation, patients were required to wear Bluetooth devices when doing physical activity, thus data like heart rate [26], respiration rate, blood oxygen saturation, and calorie consumption could be recorded by the mHealth app^a^ [22], doctors could check the exercise data then give the exercise prescription according to the FITT principle.

#### Nutritional

We issued an individualized diagnosis report for patients. The report included a seven-day dietary suggestion detailing the types and amounts of food, frequency of eating, and the amount of energy, protein, or other macro-nutrient requirements. Whey protein Solid beverage^c^ (Ainst, Beijing) and high-fat, low-sugar, high-protein solid beverage (Ainst, Beijing) were provided to patients whose PG-SGA score ≥ 4. Our dietitians also offered phone nutritional counseling weekly, designed to educate patients on maintaining their target nutritional intake.

#### Psychological

VRR training system^d^ and mHealth-based MT [27]were used to improve emotional status [28, 29]. Our treatment ward was equipped with Head-mounted glasses^e^ (PICO wireless VR glasses) with hidden near-field speakers, infrared sensors, Bluetooth gamepads, and a head motion tracking system. Each patient had a controller to interact with the virtual environment to undergo music therapy, relaxation therapy [30], mindfulness therapy [31], hypnotherapy, and other multi-scene interventions [32].

Each session lasts approximately 40 min.

i) VR Music includes immersive experiences such as “My Maple Forest,” “Sunset at Half Moon Bay,” and “Journey Through the Forests of Japan”;
ii) VR Relaxation features a collection of Chinese landscapes, international landscapes, a tour of the Australian Islands, and Australia’s Dream Beaches;
iii) VR Mindfulness encompasses sessions like “Breathing,” “Journey Through Africa,” “Flower World of Foshan,” and a tour of the Rocky Mountains;
iv) VR Sleep offers serene settings including “Malibu Beach Tour,” “Tropical Beach Tour,” “Norwegian Aurora Tour,” and “Alaskan Aurora Tour” ect.

We have more than 80 virtual scenes to choose from. Each patient had a controller to interact with the virtual environment. With the gamepads, patients also could take part in games to train their reactions. Examples were shown in supplement Table 2.

Psychologists selected suitable music and uploaded it to the mHealth app [27], including piano music, soft pop songs, pure music, classical music, and the sounds of nature [33]. During the home rehabilitation period, we advised patients to have MT via mobile phone for more than 30 min daily during home rehabilitation. The app would log the usage data.

### Assessments

Grip Strength Meter, 6-minute walk monitoring analysis system^f^, Clinical Nutrition Analyzer(AiNST-CNDS20)^g^, and scales including Nutritional Risk Screening 2002 (NRS2002), Patient-generated subjective global assessment (PG-SGA), Cancer Fatigue Scale (CFS), Distress thermometer (DT), Hospital Anxiety and Depression Scale (HADS) and European Organization for Research and Treatment of Cancer Quality-of-Life Questionnaire Core 30 (EORTC QLQ-C30) ^12, 26^ were used to evaluate changes in Physical, Nutritional and Psychological aspects.

### Statistical analysis

Statistical analysis was performed using IBM SPSS Statistics 26.0 software (IBM). Baseline characteristics were summarized using median and interquartile range (IQR) for continuous variables or number and percentage for categorical variables. A paired t-test was used to determine whether there were statistically significant changes in motor function( walking speed /distance, muscle mass and skeletal muscle ), nutrition( NRS-2002, PG-SGA, basal metabolism, protein, body mass indicator, inorganic salts, body fat ratio and total body moisture ) and psychology( Distress-Thermometer DT, Cancer Fatigue Scale CSF and Hospital Anxiety and Depression Scale, HADS ) before and after the Multi-model intervention. All scales and items of the EORTC QLQ C30 were converted to a 100-point scale. The effect of interdisciplinary intervention on QoL outcomes was assessed by multivariate regression with adjustment for baseline QoL scores. P values were reported for all paired t-tests, with a cut-off of *p* < .05 for statistical significance.

## Result

### Patients

**Fig. 1:**
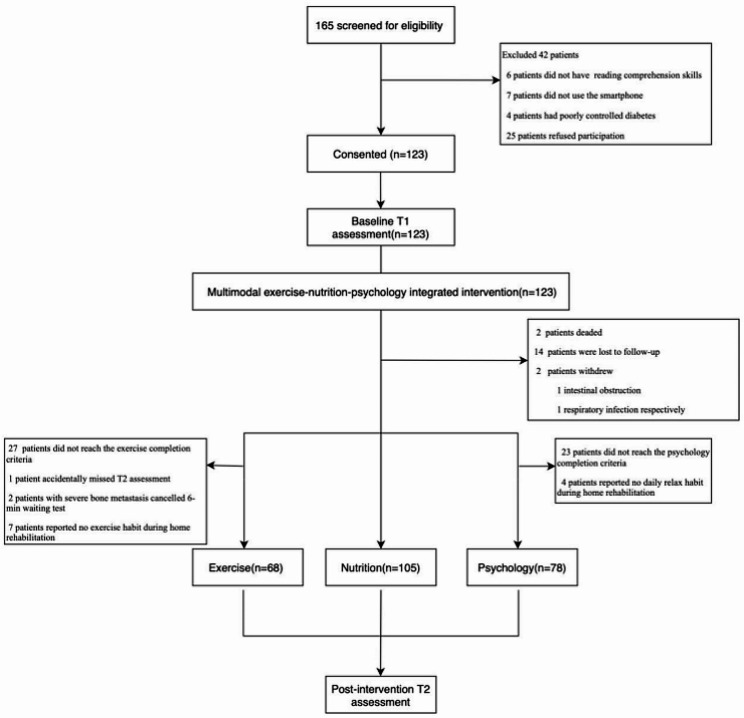
Modified CONSORT flow diagram for the single-arm Multi-modal study

Between October 2022, and April 2023, 165 cancer patients were screened and 123 were recruited (Fig. 1). Reasons for ineligibility (*n* = 42) included (i) patients did not have reading comprehension skills (*n* = 6); (ii) patients did not use the smartphone (*n* = 7); (iii) patients had poorly controlled diabetes (*n* = 4); (iv) patients declined participation to this study (*n* = 25). The median age was 62.8 years (range, 24–91 years). The majority of recruited patients were married, had a low level of education, and had a monthly income between 1,500 and 5,000 RMB; stage I ~ III cancer patients accounted for 19%, stage IV cancer patients accounted for 81%; 20% of patients had lung cancer, 19% had gastric cancer, 18% had colorectal cancer, 10% had esophageal cancer, 7% had hepatobiliary cancer, 8% had female reproductive system cancer, 6% had breast cancer, 4% had pancreatic cancer and 8% patients had tumors of other systems (Table 2).

**Table 2:** Baseline participant characteristics

*N* = 123	Range	Mean	SD
Age	24–91	62.8	11.2
	Category	N	Percentage (%)
Sex	Male	76	61%
	Female	47	39%
Cancer type	Lung cancer	25	20%
	Gastric cancer	23	19%
	Intestinal cancer	22	18%
	Esophageal cancer	12	10%
	Hepatobiliary tumor	9	7%
	Female reproductive tumor	10	8%
	Breast cancer	7	6%
	Pancreatic cancer	5	4%
	Others	10	8%
Treatment	Chemotherapy	74	60%
	Chemotherapy + Targeted therapy	14	11%
	Chemotherapy + Immunotherapy	14	11%
	Immunotherapy + Targeted therapy	7	6%
	Immunotherapy	10	8%
	Targeted therapy	2	2%
	Radiotherapy	2	2%
Cancer Staging	Stage I	5	4%
	Stage II	8	7%
	Stage III	10	8%
	Stage IV	100	81%
Educational status	Elementary School	55	45%
	Secondary School	44	36%
	High School	18	15%
	Junior College and above	6	8%
Marital status	Married	120	98%
	Unmarried	3	2%
Income (thousands/month)	Well(>¥5000)	38	31%
	General (¥1500∼¥5000)	66	54%
	Poor(<¥1500)	19	15%

### Feasibility

The recruitment rate was 75% (123/165). The completion rate of physical exercise, nutrition, and psychology were 55% (68/123), 85% (105/123), and 63% (78/123) respectively. All reached the expected endpoint. At the cutoff date (April 10, 2023), 2 (1.6%) patients died, 14 (11.4%) patients were lost to follow-up, 2 (0.81%) patients withdrew their consent due to deteriorating health status from intestinal obstruction and respiratory infection respectively unrelated to the study procedures (Fig. 1). Excluding the previous 18 patients, during the exercise intervention period, 27 patients did not reach the completion criteria (≥ 4 times participation in group-based exercise class per hospitalization). One man accidentally missed function assessments at the T2 point. The 6-minute walk test at T2 point was canceled in two patients with severe bone metastasis due to tumor progression; 7 patients reported no exercise habit (>30 min of moderate-intensity exercise per day) during the home rehabilitation period. Additionally, during VR-based psychology intervention period, 23 patients did not reach the completion criteria (≥ 4 times participation in VR therapy per hospitalization complete T1 and T2 assessments); 4 patients reported no daily MT(>30 min per day) during home rehabilitation (Fig. 1).

### Changes in physical parameters

There was no significant difference in 6-minute walking speed (*P* = .35) and grip strength (*P* = .113) in 68 patients with good exercise compliance after intervention. (Table 3).

**Table 3:** Physical function assessments

Index	T1		T2		Difference between T2 and T1 (95% CI)	*P* value
	Mean ± SD	95%CI	Mean ± SD	95%CI		
6-minute walking speed(m/s)	1.14 ± 0.2	1.10, 1.19	1.16 ± 0.2	1.11, 1.21	0.17 (-0.19, 0.53)	0.35
6-minute walking distance(m)	411.57 ± 70.34	394.54, 428.60	417.58 ± 72.15	400.12, 435.04	6.01 (-6.84, 18.85)	0.35
Handgrip strength(kg)	22.43 ± 8.53	20.37, 24.50	23.17 ± 9.41	20.89, 25.45	0.74( -1.78, 1.65)	0.113

### Changes in nutritional parameters

105 patients (105/123) completed the nutritional intervention. There were significant improvements (*P* < .05) in the mean Weight, body mass indicators(0.71 (0.51, 0.92)), NRS-2002 scores (-0.59 (-0.78, -0.40)) , PG-SGA scores (-3.305 (-3.98, -2.63)) , basal metabolism (17.59 (6.42, 28.77)) , phase angle, cell mass, body fat ratio, lean body mass, body fat, obesity degree, inorganic salts, protein (0.18 (0.07, 0.30), extracellular fluid, intracellular fluid and total body moisture from baseline to T2 time point. Their indicators of skeletal muscle, upper arm circumference, upper arm muscle circumference, upper limb muscle mass, trunk muscle mass, and total muscle mass** (**0.76 (0.26, 1.26)) were significantly improved** (***P* < .05**) **, while there was no significant difference in muscle mass of both lower limbs (*P* > .05). (Table 4)

**Table 4:** Nutrition assessment

Index	T1		T2		Difference between T2 and T1 (95% CI)	*P* value
	Mean ± SD	95%CI	Mean ± SD	95%CI		
**NRS-2002(score)**	2.65 ± 1.37	2.38, 2.91	2.06 ± 1.34	1.80, 2.32	-0.59 (-0.78, -0.40)	< 0.001^a^
**PG-SGA(score)**	8.29 ± 5.17	7.28, 9.29	4.98 ± 3.43	4.32, 5.65	-3.305 (-3.98, -2.63)	< 0.00^1a^
**Weight(kg)**	59.55 ± 12.04	57.22, 81.88	61.49 ± 11.8	59.20, 63.77	1.94 (1.42, 2.45)	< 0.001^a^
**BMI(kg/m2)**	21.93 ± 4.03	21.15, 22.71	22.64 ± 3.94	21.88, 23.40	0.71 (0.51, 0.92)	< 0.001^a^
**Basal metabolism(kcal)**	1351.42 ± 173.19	1317.90, 1384.93	1369.01 ± 176.16	1334.92, 1403.10	17.59 (6.42, 28.77)	0.002^a^
**Whole-body phase angle (°)**	5.61 ± 0.84	5.45, 5.78	5.86 ± 0.86	5.69, 6.02	0.244 (0.12, 0.37)	< 0.001 ^a^
**Body cell volume(kg)**	29.21 ± 5.34	28.18, 30.25	29.83 ± 5.39	28.78, 30.87	0.61 (0.22, 1.00)	0.002 ^a^
**Body fat (kg)**	14.06 ± 7.42	12.62, 15.49	15.24 ± 7.85	13.72, 16.76	1.19 (0.56, 1.81)	< 0.001 ^a^
**Body fat rate(%)**	22.68 ± 9.27	20.89, 24.47	23.91 ± 9.89	22.00, 25.82	1.23 (0.23, 2.24)	0.016 ^a^
**lean body mass(kg)**	45.44 ± 8.02	12.62, 15.49	46.25 ± 8.16	44.67, 47.83	0.81 (0.30, 1.33)	0.002 ^a^
**Visceral fat area(cm2)**	70.77 ± 37.93	63.43, 78.11	76.11 ± 43.39	67.72, 84.51	5.35 (0.55, 10.15)	0.029 ^a^
**Obesity degree(%)**	103.16 ± 18.96	99.49, 106.83	106.56 ± 18.53	102.97, 110.14	3.40 (2.44, 4.37)	< 0.001 ^a^
**Inorganic salts(kg)**	3.12 ± 0.48	3.02, 3.21	3.18 ± 0.5	3.08, 9.32	0.063 (0.02, 0.11)	0.007 ^a^
**Protein(kg)**	8.82 ± 1.61	8.51, 9.13	9 ± 1.63	8.69, 9.32	0.18 (0.07, 0.30)	0.003 ^a^
**Total body water(L)**	33.5 ± 5.96	32.34, 34.65	34.07 ± 6.08	32.90, 35.25	0.57 (0.19, 0.96)	0.004 ^a^
**Intracellular water(L)**	15.77 ± 4.63	14.88, 16.67	16 ± 4.83	15.07, 16.94	0.23 (0.03, 0.43)	0.024 ^a^
**Extracellular water(L)**	17.73 ± 4.75	16.81, 18.65	18.07 ± 4.84	17.13, 19.00	0.34 (0.12, 0.58)	0.005 ^a^
Skeletal muscle mass(kg)	24.6 ± 4.86	23.66, 25.55	25.15 ± 4.9	24.20, 26.10	0.55 (0.19, 0.90)	0.003 ^a^
Upper arm circumference (cm)	28.42 ± 3.99	27.65, 29.19	24.37 ± 2.7	23.85, 24.90	0.88 (0.59, 1.18)	< 0.001 ^a^
Upper arm muscle circumference (cm)	23.73 ± 3.31	23.09, 24.37	24.37 ± 2.7	23.85, 24.90	0.64 (0.30, 0.98)	< 0.001 ^a^
Upper limb muscle mass(left)(kg)	2.34 ± 0.61	2.23, 2.46	2.42 ± 0.65	2.29, 2.54	0.07 (0.33, 0.11)	< 0.001 ^a^
Upper limb muscle mass (right)(kg)	2.42 ± 0.65	2.30, 2.55	2.53 ± 0.65	2.40, 2.66	0.11 (0.68, 0.15)	< 0.001 ^a^
Trunk muscle mass(kg)	20.29 ± 3.85	19.54, 21.03	20.81 ± 3.92	20.05, 21.57	0.53 (0.30, 0.75)	< 0.001 ^a^
Total muscle mass(kg)	42.87 ± 7.66	41.49, 44.35	43.63 ± 7.79	42.12, 45.14	0.76 (0.26, 1.26)	0.003 ^a^
Lower limb muscle mass(left)(kg)	6.84 ± 1.51	6.55, 7.13	6.97 ± 1.49	6.68, 7.26	0.13 (-0.01, 0.26)	0.063
Lower limb muscle mass (right) (kg)	6.90 ± 1.55	6.60, 7.20	7.02 ± 1.53	6.73, 7.32	0.122 (-0.17, 0.26)	0.084

*Abbreviations* NRS-2002, nutrition risk screening-2002; PG-SGA, patient-generated subjective global assessment.a = *p* < .05

### Changes in psychological parameters

**Fig. 2:**
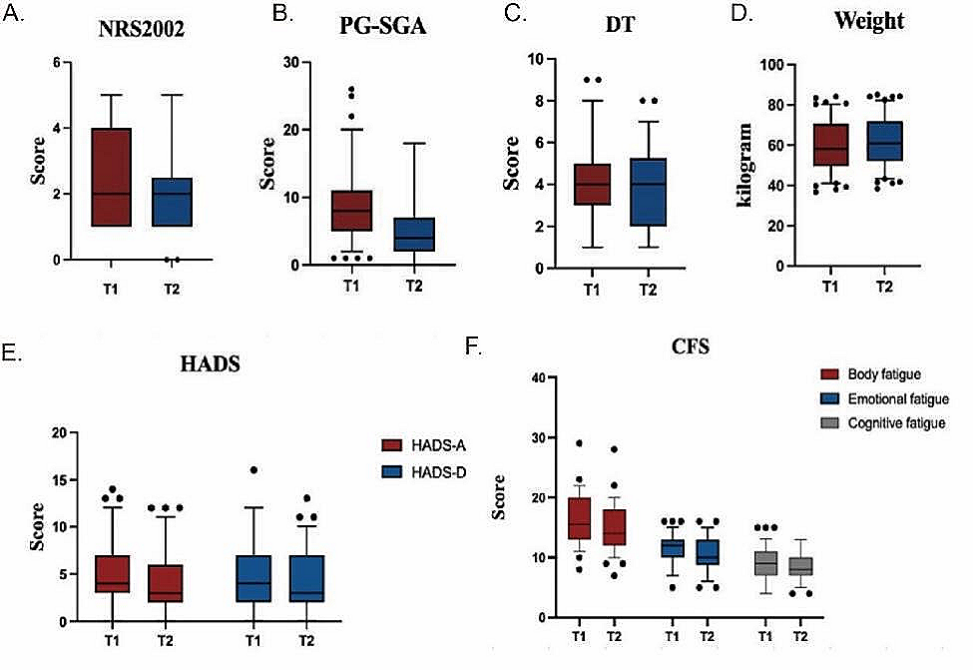
(**A**) Boxplots for NRS2002 scale scores. Significant improvements were observed in the mean score from baseline to week 8 in NRS 2002 (2.65, 95% CI, 2.38 to 2.91, v 2.06, 95% CI, 1.80 to 2.32, *P* < .001). (**B**) Boxplots for PG-SGA scales, PG-SGA (8.29, 95% CI,7.28 to 9.29, v 4.98, 95% CI,4.32 to 5.65, *P* < .001). (**C**) Boxplots for DT (4.24, 95% CI, 3.79 to 4.69, v 3.86, 95% CI, 3.40 to 4.32, *P* = .005). (**D**) Boxplots for Weight (59.55, 95% CI, 57.22 to 81.88, v 61.49, 95% CI, 59.20 to 63.77, *P* < .001). (**E**) Boxplots for HADS. Significant improvements were observed in the mean score from baseline to week 8 in HADS-A (4.76, 95% CI, 4.03 to 5.49, v3.95, 95% CI, 3.24 to 4.65, *P* < .001), HADS-D (5.01, 95% CI, 4.18 to 5.85, v4.14, 95% CI, 3.24 to 4.86, *P* < .001). (**F**) Boxplots for CFS scales, Body Fatigue (16.15, 95% CI, 15.27 to 17.03, v14.85, 95% CI, 14.02 to 15.68, *P* < .001) and Emotional Fatigue(11.58, 95% CI, 11.04 to 12.11, v10.63, 95% CI, 10.03 to 11.23, *P* = .007),while in the aspect of Cognitive fatigue there was no significant improvements, Cognitive fatigue(9.10, 95% CI, 8.47 to 9.74, v8.53, 95% CI, 8.04 to 9.04, *P* = .107)

78 patients completed the psychological intervention, and we observed a significant drop in mean scores from baseline (T1) to week 8 (T2) on the DT, Hospital Anxiety and Depression Scale-Anxiety (HADS-A), Hospital Anxiety and Depression Scale-Depression (HADS-D), and CSF-Body fatigue and CSF-Emotional fatigue scales. (Table 6; Fig. 2). The results showed significant improvements in Mean quality-of-life scores (Table 5), except for the aspect of role functioning (Table 5).

**Table 5:** EORTC QLQ-C30 (version 3) **Abbreviations: EORTC QLQ-C30, European Organization for Research and Treatment of Cancer Quality-of-Life Questionnaire Core**

Functioning area	T1		T2		Difference between T2 and T1 (95% CI)	*P* Value
	Mean ± SD	95%CI	Mean ± SD	95%CI		
Physical Functioning( PF)	64.96 ± 20.03	60.44, 69.47	69.57 ± 20.46	64.96, 74.19	4.62 (3.30, 5.93)	< 0.001a
Role Functioning( RF)	68.59 ± 23.57	63.28 ,73.90	66.67 ± 22.95	61.49 ,71.84	-1.92 (-4.63, 0.78)	0.161
Emotional Functioning( EF)	76.18 ± 19.63	71.75, 80.60	80.56 ± 18.83	76.31, 84.80	4.38 (2.79, 5.98)	< 0.001a
Cognitive Functioning( CF)	77.99 ± 18.31	73.86 ,82.12	81.41 ± 18.01	77.35, 85.47	3.42 (0.97, 5.87)	0.007a
Social Functioning( SF)	65.38 ± 20.24	60.82, 69.95	70.94 ± 22.22	65.93, 75.95	5.56 (2.94, 8.17)	< 0.001a
Global Health Status( QL)	60.47 ± 20.61	55.82, 65.12	66.45 ± 21.45	61.62, 71.29	5.98 (3.88 ,8.08)	< 0.001a

**Table 6:** Psychological assessment scales

Category	Scale	T1		T2		Difference between T2 and T1 (95% CI)	*P* Value
		Mean ± SD	95%CI	Mean ± SD	95%CI		
HADS	HADS-A	4.76 ± 3.24	4.03, 5.49	3.95 ± 3.12	3.24, 4.65	-0.81(-1.05, -0.57)	< 0.001a
	HADS-D	5.01 ± 3.72	4.18, 5.85	4.14 ± 3.19	3.24, 4.86	-0.87(-1.17, -0.58)	< 0.001a
DT	DT	4.24 ± 2	3.79, 4.69	3.86 ± 2.03	3.40, 4.32	-0.38(-0.65, -0.12)	0.005a
CFS	Body fatigue	16.15 ± 3.9	15.27, 17.03	14.85 ± 3.68	14.02, 15.68	-1.31(-1.68, -0.93)	< 0.001a
	Emotional fatigue	11.58 ± 2.36	11.04, 12.11	10.63 ± 2.65	10.03, 11.23	-0.95(-1.63, -0.27)	0.007a
	Cognitive fatigue	9.10 ± 2.82	8.47, 9.74	8.53 ± 2.20	8.04, 9.04	-0.56(-1.25, 0.12)	0.107a

*Abbreviations* HADS, Hospital Anxiety and Depression Scale; HADS-A, Hospital Anxiety and Depression Scale-Aniexity; HADS-D, Hospital Anxiety and Depression Scale-Depression; CFS, Cancer Fatigue Scale; DT, Distress thermometer. a = *p* < .05

**Table 5:** EORTC QLQ-C30 (version 3)

Functioning area	T1		T2		Difference between T2 and T1 (95% CI)	*P* Value
	Mean ± SD	95%CI	Mean ± SD	95%CI		
Physical Functioning( PF)	64.96 ± 20.03	60.44, 69.47	69.57 ± 20.46	64.96, 74.19	4.62 (3.30, 5.93)	< 0.001a
Role Functioning( RF)	68.59 ± 23.57	63.28 ,73.90	66.67 ± 22.95	61.49 ,71.84	-1.92 (-4.63, 0.78)	0.161
Emotional Functioning( EF)	76.18 ± 19.63	71.75, 80.60	80.56 ± 18.83	76.31, 84.80	4.38 (2.79, 5.98)	< 0.001a
Cognitive Functioning( CF)	77.99 ± 18.31	73.86 ,82.12	81.41 ± 18.01	77.35, 85.47	3.42 (0.97, 5.87)	0.007a
Social Functioning( SF)	65.38 ± 20.24	60.82, 69.95	70.94 ± 22.22	65.93, 75.95	5.56 (2.94, 8.17)	< 0.001a
Global Health Status( QL)	60.47 ± 20.61	55.82, 65.12	66.45 ± 21.45	61.62, 71.29	5.98 (3.88 ,8.08)	< 0.001a

*Abbreviations* EORTC QLQ-C30, European Organization for Research and Treatment of Cancer Quality-of-Life Questionnaire Core 30

### Safety

From the beginning of the intervention to 4 weeks after the end, safety was assessed for all 123 patients. Adverse events possibly related to the mHealth and VR-based exercise-nutrition-psychology rehabilitation program were observed in 3 patients, including 1 patient who reported dyspnea during exercise and 2 patients who reported dizziness. During the intervention, 2(1.6%) patients died due to multi-organ failure and cerebral infractions, 1 (0.4%) patient reported intestinal obstruction and 1 patient experienced respiratory infection. These were unrelated to the rehabilitation program.

## Discussion

Nowadays, there is a lack of comprehensive rehabilitation guidelines, and the existing guidelines for cancer survivors mainly just deal with exercise or nutrition. Our study first explores the feasibility of a VR and mHealth based exercise-nutrition-psychology integrated rehabilitation model in China. This feasibility study meets the expected recruitment (70%) and compliance rate (50%), showing an adequate safety profile and a reasonable dropout. Patients complied well with the requirements for group-based physical activity, nutritional counseling, MT, VR treatment, and mHealth-based tracking.

Maintaining motivation and adherence to nutritional or exercise interventions in cancer patients is particularly challenging [34]. Baldwin et al. reported detailed compliance data in a large randomized controlled trial focused on nutritional intervention for patients with advanced cancer and weight loss. Initially, 25% of patients completed food diaries and 31% consumed supplements, but these figures fell to 17% and 19%, respectively, after six weeks. The study was terminated early due to lack of efficacy, highlighting that poor compliance may limit the intervention’s effectiveness [35]. Zhihao Lu et al. conducted a bi-intervention study combining nutrition and psychology in previously untreated patients with metastatic esophagogastric cancer, reporting detailed compliance data. The dropout rate was 33% for nutritional interventions and 28% for psychological interventions [23].

Exercise interventions for cancer patients face challenges with low recruitment, high dropout rates, and inconsistent adherence. Reynolds ect. analyzed 87 exercise intervention trials and found the median recruitment rate for all trials was 38% (range 0.52–100%) (mean 42.96%) [36]. Common reasons for non-participation included lack of interest (46.51%, n (number of studies) = 40); distance and transport (45.3%, n (number of studies) = 39); and failure to contact (44.2%, n (number of studies) = 38). A feasibility study of exercise interventions for patients with pancreatic and non-small-cell lung cancer [37] reported only 21% patients reached the target steps for exercise.

We tried to maximize adherence without compromising the effectiveness of the intervention. First, we provided detailed meal plans and promptly supplied oral nutritional supplements to malnourished patients, enabling them to quickly experience the benefits of nutritional interventions. Second, we used an mHealth app to monitor patients’ exercise intensity in real-time, assess their current health status, issue exercise prescriptions, and remind patients to complete daily music therapy. For patients (12/123) whose data was not collected by the mHealth app, we conducted weekly phone follow-ups as an alternative recording method. During the 8-week study period, that compliance with nutrition is the highest, followed by VR and mHealth-based psychology. Significant improvements were observed in both psychological and nutritional functions among our patients, In terms of physical function, although there were increases in the average values of grip strength and the 6-minute walk distance, neither showed significant improvement. This lack of significant improvement may be attributed to the short intervention period and the ongoing radiotherapy or chemotherapy during the intervention [38, 39]. Although the short-term intervention did not significantly enhance physical function, our data indicated a protective increase effect on total muscle mass, suggesting no deterioration.

Our study has several limitations. This study is a single-center feasibility study, and the study population is not heterogeneous in terms of cancer type and treatment regimen; There were differences in some indicators but no statistical significance, so it was necessary to expand the sample size for further verification; Some wearable devices (Bluetooth armbands) are not portable and need to be further improved. The exercise prescription software in this study is still in its early version and needs to be optimized; Due to the current limited sample size, we are temporarily unable to conduct a detailed analysis based on tumor type and disease severity.

In future multicenter clinical trials, we will expand the sample size to enhance the reliability and comprehensiveness of our data analysis. This will enable us to conduct more detailed comparative studies on different types of tumors and varying levels of disease severity. In terms of content optimization, we plan to further enhance patient education, increase guidance and follow-up during home rehabilitation, and improve adherence to psychological and exercise interventions. Additionally, we will investigate the efficacy of personalized, long-term exercise interventions, particularly targeting hand strength training and cognitive fatigue [40].

## Conclusion

In conclusion, it is feasible to conduct mHealth and VR-based nutrition-exercise-psychology rehabilitation study in cancer patients, such a model shows potential benefits in terms of nutrition, emotion, and muscle mass improvements. Our study provides new clinical evidence and lays the foundation for the application of new tech–mHealth and VRR in cancer rehabilitation. The mHealth is an effective method to collect and analyze patients’ data and improve the efficiency of follow-up visits. Both VR and mHealth-based MT are useful interventions for alleviating anxiety and improving mood states. Further investigation in a larger cohort trial is warranted. In the future, with the update of technology, VR and mHealth will have a wider application in the field of cancer rehabilitation.

### Electronic supplementary material

Below is the link to the electronic supplementary material.

Supplementary Material 1

Supplementary Material 2

## Data Availability

Data will be made available on reasonable request, all data sharing and collaboration requests should be directed to the corresponding author HJ( czeyjh@njmu.edu.cn).

## Abbreviations

App: Application
BMI: Body mass indicator
CFS: Cancer Fatigue Scale
DT: Distress thermometer
EORTC QLQ-C30: European Organization for Research and Treatment of Cancer
HADS: Hospital Anxiety and Depression Scale
mHealth: mobile health
METs: metabolic equivalents
MT: music therapy
NRS2002: Nutritional Risk Screening 2002
PG-SGA: Patient-generated subjective global assessment
Quality-of-Life: Questionnaire Core 30
VR: Virtual reality
VRR: Virtual Reality Rehabilitation
