# Supplementary material for: Feasibility of an exercise-nutrition-psychology integrated rehabilitation model based on mobile health and virtual reality for cancer patients: a single-center, single-arm, prospective phase II study
Source: BMC Palliat Care. 2024 Jun 20;23:155. doi: 10.1186/s12904-024-01487-3 (PMC11191250; doi:10.1186/s12904-024-01487-3)
Supplement: Supplementary file 1 — Supplementary Material 1 [file 12904_2024_1487_MOESM1_ESM.docx]

**Supplement Table 1.**

| Training phase | Action | Frequency | Training type | Location | Training equipment | Notes |
| --- | --- | --- | --- | --- | --- | --- |
| Warming up  phase | Jump in place | 8 X 2 | Cardiopulmonary | Whole body | Bare-handed |  |
|  | Side ski step | 8 X 2 | Cardiopulmonary | Whole body | Bare-handed |  |
| Intermittent | 5s | | | | |  |
| General training phase | Horizontal rotation of the body in the standing position | 8 X 2 | Stretching | Spinal column | Bare-handed |  |
|  | Bend over and rotate your torso to touch your toes | 8 X 2 | Cardiopulmonary | Whole body | Bare-handed |  |
|  | Standing leg raises | 30 X 2 | Cardiopulmonary | Whole body | Bare-handed |  |
|  | Standing in place alternating shoulder presses and high leg raises | 8 X 2 | Cardiopulmonary | Whole body | Bare-handed |  |
|  | Standing alternating elbows and knees touching | 30 X 2 | Cardiopulmonary | Whole body | Bare-handed |  |
| Intermittent | 5s | | | | |  |
| Organizational phase | Front kick with one leg standing (left) | 8 X 2 | Strength | Lower extremities | Bare-handed |  |
|  | Front kick with one leg standing (right) | 8 X 2 | Strength | Lower extremities | Bare-handed |  |
|  | Side kick (left) holding the wall with one leg standing | 8 X 2 | Strength | Lower extremities | Bare-handed | The patient can hold chair backs or headboards to ensure that there is a support to control balance |
|  | Side kick (right) holding the wall with one leg standing | 8 X 2 | Strength | Lower extremities | Bare-handed |  |
| Intermittent | 5s | | | | |  |
| Stretching phase | Bend over and stretch the calf triceps(left） | 30s X 1 | Stretching | Calves | Bare-handed |  |
|  | Bend over and stretch the calf triceps (right) | 30s X 1 | Stretching | Calves | Bare-handed |  |
|  | Stretch the pectoralis major muscle | 30s X 2 | Stretching | Chest | Bare-handed |  |
| Repeat three times | | | | | |  |
