# Supplementary material for: Feasibility of an exercise-nutrition-psychology integrated rehabilitation model based on mobile health and virtual reality for cancer patients: a single-center, single-arm, prospective phase II study
Source: BMC Palliat Care. 2024 Jun 20;23:155. doi: 10.1186/s12904-024-01487-3 (PMC11191250; doi:10.1186/s12904-024-01487-3)
Supplement: Supplementary file 2 — Supplementary Material 2 [file 12904_2024_1487_MOESM2_ESM.docx]

Supplement table2

| Title | Visuals | Brief introduction |
| --- | --- | --- |
| My Maple Forest | 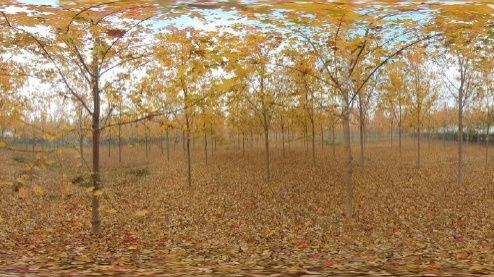 | I walk among the maple trees, seeing the ground covered in red and yellow maple leaves. I pick up a red maple leaf, smaller than my palm but richly colored, as red as fire. |
| Collection of Chinese Landscapes | 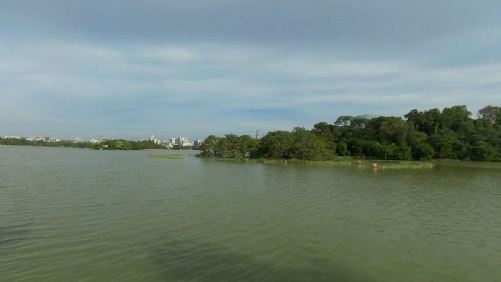 | The lake is beautiful. With spring returning to the land, walking along the lake’s embankments and looking at the calm lake surface creates ripples that touch the soul. Willow branches gently brush your cheeks, whispering over your head and shoulders. The distant, enchanting sound of a guqin plays, intoxicating the listener. |
| Breathing | 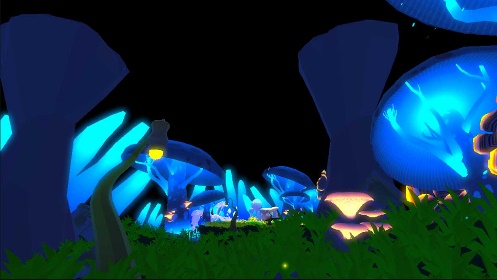 | "Breathing" is a VR application designed to help users relax. It guides users through adjusting their breathing rhythm with soothing music, beautiful environments, and guided prompts, achieving stress relief and promoting sleep. |
| Malibu Beach Tour | 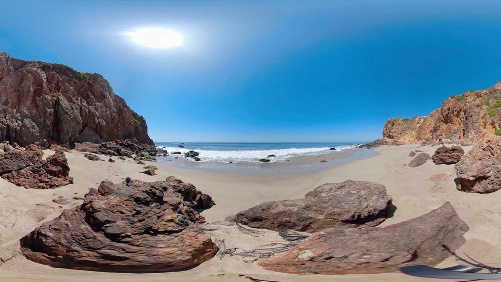 | Gaze at the endless ocean and listen to the waves crashing on the beach. This VR application guides users through adjusting their breathing rhythm with soothing music, the beautiful vistas of Malibu Beach, and guided prompts, achieving stress relief and promoting sleep. |
